# Verification of Finger Joint Stiffness Estimation Method With Soft Robotic Actuator

**DOI:** 10.3389/fbioe.2020.592637

**Published:** 2020-12-18

**Authors:** Xiang Qian Shi, Ho Lam Heung, Zhi Qiang Tang, Kai Yu Tong, Zheng Li

**Affiliations:** ^1^Department of Biomedical Engineering, The Chinese University of Hong Kong, Shatin, Hong Kong; ^2^Department of Surgery, The Chinese University of Hong Kong, Shatin, Hong Kong

**Keywords:** soft-elastic composite actuator (SECA), SECA-finger modeling, passive joint stiffness, metacarpophalangeal joint, stroke, spasticity

## Abstract

Stroke has been the leading cause of disability due to the induced spasticity in the upper extremity. The constant flexion of spastic fingers following stroke has not been well described. Accurate measurements for joint stiffness help clinicians have a better access to the level of impairment after stroke. Previously, we conducted a method for quantifying the passive finger joint stiffness based on the pressure-angle relationship between the spastic fingers and the soft-elastic composite actuator (SECA). However, it lacks a ground-truth to demonstrate the compatibility between the SECA-facilitated stiffness estimation and standard joint stiffness quantification procedure. In this study, we compare the passive metacarpophalangeal (MCP) joint stiffness measured using the SECA with the results from our designed standalone mechatronics device, which measures the passive metacarpophalangeal joint torque and angle during passive finger rotation. Results obtained from the fitting model that concludes the stiffness characteristic are further compared with the results obtained from SECA-Finger model, as well as the clinical score of Modified Ashworth Scale (MAS) for grading spasticity. These findings suggest the possibility of passive MCP joint stiffness quantification using the soft robotic actuator during the performance of different tasks in hand rehabilitation.

## Introduction

Spasticity, commonly known as a symptom with a broad range of neurological disorders, is frequently found in subjects after stroke. Previous studies indicated that about 40% of subjects with stroke suffer from spasticity (Francisco and McGuire, 2012; Huang and Patton, 2016), leading to a huge burden on those subjects and challenges to the nursing staff (Yi et al., 2013). Finger flexor spasticity, usually characterized by hyper-resistance in the finger joint, which is a result of pathological neuromuscular activation and biomechanical changes in muscles and soft tissues overlying the joint. Distribution and level of these neural and non-neural components may diverge between individual subjects, and it may change during the time course of post stroke (Andringa et al., 2019). In other words, this spasticity increases stiffness of the finger joints and furthermore leads to a decrease of their range-of-motion (ROM), creating severe reduction to hand function (Sadarangani et al., 2017). To treat subjects with spasticity, several different approaches such as local botulinum toxin injection, physical and occupational therapies, electrical neuro stimulation, and surgical interventions, have been commonly used in clinic. Despite those approaches have shown their effectiveness in clinical spasticity treatments, the mechanisms that underlie and the influences of spasticity on functional movement are still not well understood. Previous clinical studies rarely focused on the changes of passive finger joint stiffness opposing the joint rotation, though it may reflect the spasticity of the finger flexors (Kamper and Rymer, 2000). Hence, management of spasticity includes reliable and accurate assessments of passive finger joint stiffness is needed for better identification of treatment strategies and goals.

However, the only assessments regularly incorporated into neurorehabilitation are through clinical measures (Fugl-Meyer et al., 1975; Gregson et al., 1999), which are typically subjective, labor intensive, and graded on an ordinal scale. Nevertheless, result subjectivity and rater reliability have been continuously questioned by researchers since the measurement is dependent on clinicians’ experience (Damiano et al., 2002; Fleuren et al., 2010). Modified Ashworth Scale (MAS), for example, the most popularly used scale has a relatively simple protocol (Blackburn et al., 2002; Mehrholz et al., 2005). Junior clinicians learn the MAS from the instruction first and develop their own standard through their clinical experiences (Park et al., 2017). There are no common standards to train the clinicians. Therefore, it would be satisfied to propose an objective and effective way to assess the spasticity of finger flexors. In contrast, robotic measures offer the possibility for objective, efficient, and descriptive assessments (Lambercy et al., 2012; Maggioni et al., 2016).

The most common clinical examination of spasticity includes assessment of exaggerated tendon tap reflexes by passive muscle stretch. Several rehabilitation instruments have been built in laboratories to quantitatively examine joint resistance to passive stretch. The evaluation of biomechanical joint properties (Nalam and Lee, 2019), such as passive joint stiffness and the range of motion (ROM), is hopefully achieved through an instrument-aided assessment. Rehabilitation robot commonly provides a relatively objective and stable platform for the analysis on the biomechanical behavior of fingers for subjects with stroke and no neurological deficit. Many clinical studies have investigated the passive joint stiffness of the index finger metacarpophalangeal (MCP) joint, due to its prominent role in many hand functions (Minami et al., 1983; Esteki and Mansour, 1996; Kamper et al., 2002; Kuo and Deshpande, 2012). Various mechatronic devices are thereby designed for standalone passive finger joint stiffness measurement after stroke (Milner and Franklin, 1998; Kamper et al., 2006; Yap et al., 2016). These measurements focus on the changes in passive stiffness opposing MCP rotation to further quantify the spasticity of finger flexors. However, due to the bulky size of all components, mechatronic devices are hard to be used to examine the stiffness of other joints across different fingers, e.g., proximal interphalangeal (PIP) joints that the stiffness is comparable to MCP joints after contracture (Prosser, 1996; Tang et al., 2019), in each finger. In addition, current measurements are separated from activities of daily living (ADL) and therapeutic training. Regular joint stiffness measurement as an indication of the performance of finger function during rehabilitation would be ineffective, and thereby only pre-determined training exercises could be offered to subjects regardless of their joint stiffness condition (Kamper et al., 2006). Continuous assessment of finger stiffness would be helpful and valuable to provide information for physiotherapists to adjust the rehabilitation models and formulate timely and accurate treatment plans that are suitable to subjects’ condition (Kamper et al., 2006).

Previously, we introduced the first 3D printed soft-elastic composite actuator (SECA) for hand rehabilitation that quantifies stiffness of the impaired fingers (Heung et al., 2020). We further demonstrated the methodology of applying this soft actuator to finger stiffness evaluation using its static models. The accuracy of the models was validated both in free space bending and on phantom fingers. Experimental results showing the joint stiffness of subjects with chronic stroke and no neurological deficit were obtained using the static model, which are supportive to existing clinical measures. However, validation of our method feasibility and compatibility in our previous study (Heung et al., 2020) only involved experiments performed on mannequin hands with different torsion springs with preset stiffness values. It would be necessary to compare the results obtained from our new method with existing ground-truth joint stiffness values based on the well-validated stiffness quantification methods in existing literatures.

In this work, we replicated the standalone stiffness measurement device proposed by Esteki and Mansour (1996) and Kuo and Deshpande (2012) for measuring the passive joint moment and angle for the MCP joint in the index finger. Ground-truth values of MCP joint stiffness would be obtained by taking derivative of the double exponential function-based model for finger kinematics (Esteki and Mansour, 1996; Silder et al., 2007; Deshpande et al., 2011) that fitted with collected joint torque and angle data, which has been the standard procedures in existing literatures of presenting the torque-angle relationship (Milner and Franklin, 1998; Kamper et al., 2006; Yap et al., 2016). Previous studies already showed that torque required to extend the hypertonic MCP joint was nearly linear with respect to the joint angle, thus showing an almost constant rotational stiffness as soon as the effect of joint capsule-ligament complex (CLC) that would increase the passive resistance upon closing to full flexion or extension was not considered (Kamper and Rymer, 2000; Kamper et al., 2003; Kuo and Deshpande, 2012). Accuracy of the mechatronics device we replicated, and the stiffness quantification method, was also already demonstrated in their study with their thirteen subjects (Esteki and Mansour, 1996) and (Kuo and Deshpande, 2012). In our study, eight subjects (four subjects with chronic stroke and four subjects with no neurological deficit) are recruited for the evaluation of MCP joint stiffness in their index fingers using our method and existing standardized method. Results of MCP joint stiffness are compared with each other, as well as the MAS scores, for reflecting the clinical potential of our method. This effort is undertaken with the intent to improve our understanding of the change of finger stiffness during among subjects with different levels of impairment after stroke in an objective manner, together with the expectation of assisting in the guidance of more effective hand rehabilitation training in the future.

### SECA-Finger Modeling for Stiffness Evaluation

#### System Description

**FIGURE 1:**
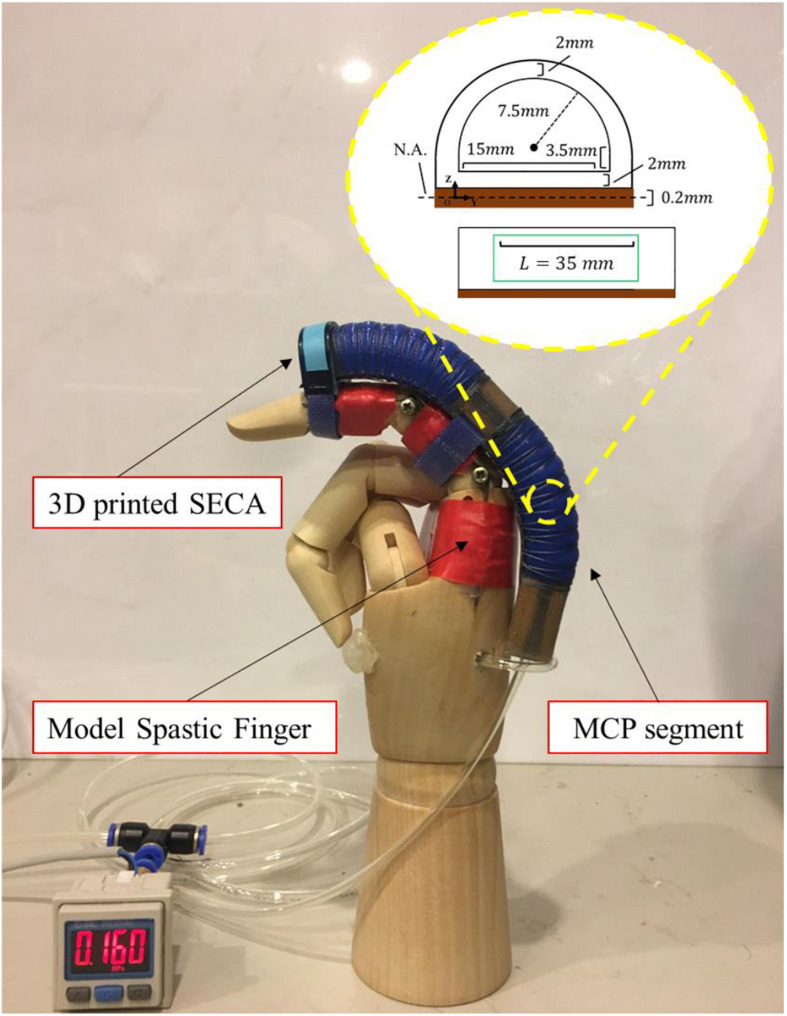
Demonstration of actuated movement of the 3D printed SECA on a model spastic finger. Example actuated state of the MCP joint bound with the MCP segment of the SECA at 160 kPa pressure. Size of the MCP segment is labeled in the figure.

We have designed the 3D printed SECA that can actively control flexion and extension of a spastic finger. MCP segment on the SECA corresponds to actuation of the MCP joint of the spastic finger, which joint flexion is controlled on pressurization and extension is controlled on depressurization (Heung et al., 2019). Flex sensor (Flex-point Sensor System, Draper, UT, United States) is placed beneath the MCP segment for measuring the bending angle. Besides, we have developed the SECA-Finger model for predicting the bending performance of SECA on spastic finger, as illustrated in Figure 1 (Tang et al., 2019; Heung et al., 2020). The influence of spastic fingers to SECA actuation is also addressed by the model, allowing us to obtain indicative results regarding the stiffness of MCP joint.

#### Static Modeling

Previously, we have already derived the SECA-Finger model that presents the bending performance of SECA on a spastic finger (Heung et al., 2020). As only the joint stiffness due to flexor tone is of our interest, we only consider the joint angle range between the fully extended position and the initial resting position (i.e., θ≥0 and θ < θ_0_, θ is the bending angle that θ_*m*_ is for the current bending angle of MCP joint and θ_*0*_ is the resting angle of the joint and always less than 90°). To demonstrate the pressure-angle relationship, we have obtained the following equation of (Heung et al., 2020)

(1) $$P=\frac{2w_{m}aL^{2}\left(a+\pi \left(a+b\right)+2r\right)+EI\theta^{2}-kL{\left(\theta -\theta_{0}\right)}^{2}}{2L\left(\frac{\pi }{2}\left(\frac{t}{2}+a+b\right)r^{2}+eb\left(\frac{t}{2}+a\right)+\frac{eb^{2}}{2}+\frac{2r^{3}}{3}\right)\theta }$$

in which *a* is the actuator wall thickness, *b* is the actuator internal rectangular height, *e* is the actuator internal chamber width, *r* is the actuator internal circular radius, *t* is the layer thickness, *L* is the actuator internal chamber length (*L*_*m*_ for MCP segment), *w*_*m*_ is the actuator strain energy density function, *P* is the input pressure, *E* is the layer Young’s modulus, *I* is the layer second moment of area, *k* is the joint stiffness (*k*_*m*_ for MCP joint), θ is the joint angle (θ_*m*_ for MCP joint, in radian) (Heung et al., 2020).

Rearranging the analytical model of equation (1) in the region of θ ∈ [0^0^,θ_0_], the joint stiffness equation is

(2) $$k_{m}=\frac{A+EI\theta_{m}^{2}-B\theta_{m}}{L{\left(\theta -\theta_{0}\right)}^{2}}$$

which

$$\begin{array}{c} A=2w_{m}aL^{2}\left(a+\pi \left(a+b\right)+2r\right)\\ B=2PL\left(\frac{\pi }{2}\left(\frac{t}{2}+a+b\right)r^{2}+eb\left(\frac{t}{2}+a\right)+\frac{eb^{2}}{2}+\frac{2r^{3}}{3}\right) \end{array}$$

Furthermore, γ is an empirical coefficient defined for MCP joint angle. The value is set to be 0.7 to avoid reaching singularity (i.e., only small difference between the bending angle of SECA θ and the resting angle of finger joint θ_*0*_) and generating inaccurate results. Therefore, the possible ranges of MCP joint angle and input pressures for stiffness evaluation using equation (2) become

(3) $$\theta_{m}\in \left[\theta_{i\_m},\gamma \theta_{0\_m}\right],0<\gamma <1$$

(4) $$P=cutoff,when\left(\theta_{m}>\gamma \theta_{0\_m}\right)$$

where θ_*i_ m*_ is the extended MCP joint angle upon wearing of the SECA in a unpressurized state (i.e., *P = 0* and θ_*i_ m*_ > 0, since the SECA would not be able to fully extend the spastic MCP joint, as discussed previously when we were presenting the principle of SECA; Heung et al., 2020), θ_*0_ m*_ is the resting angle of MCP joint, γθ_0_*m*_ is the upper limit of MCP joint angle.

Resting angle of MCP joint θ_*0_ m*_ with bare hand only is measured prior to the experiment. Then, the ROM for stiffness evaluation begins from the initial extended MCP joint angle facilitated by the SECA. The end of ROM is set as 70% of the resting angle θ_*0_ m*_, as we previously discovered that the range between θ_*i_ m*_ and γθ_0_*m*_ (γ = 0.7) would provide the most stable stiffness results for finger joints and prevent model singularity (Heung et al., 2020). Here, γ = 0.7 was established as an empirical value from our previous research work for the upper limit of MCP joint angle (Heung et al., 2020). As soon as the measured MCP joint angle exceeds this limit, the SECA will not be further actuated. Eventually, the average of all the stiffness values calculated at different pressure points is the final MCP joint stiffness measured by the SECA.

### Ground-Truth Stiffness Evaluation With Standard Mechatronic Devices

#### System Description

**FIGURE 2:**
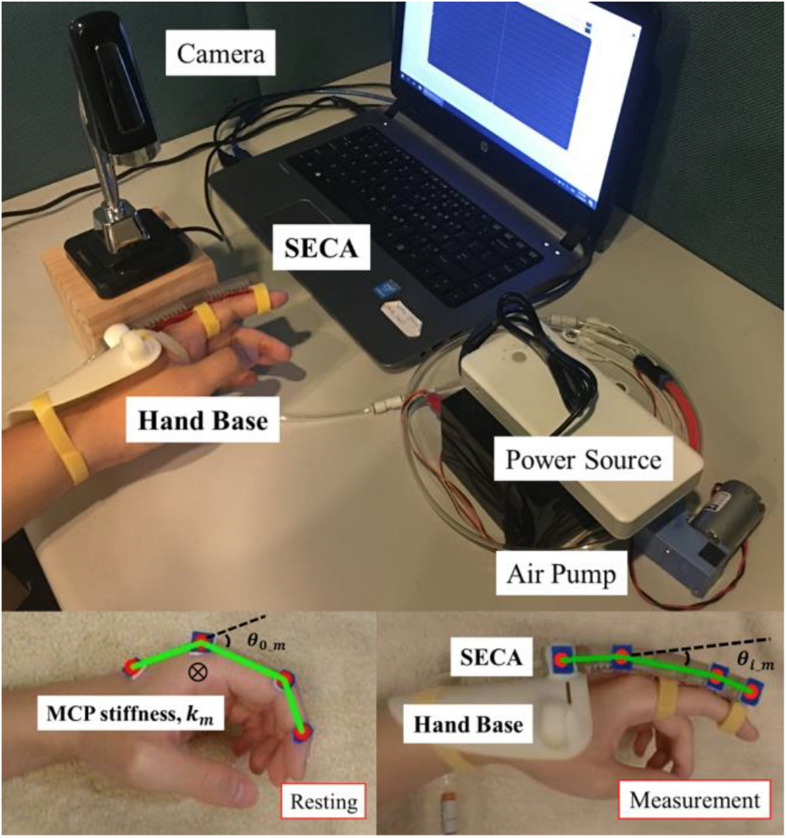
Attachment of the SECA to finger with the hand base during MCP joint stiffness measurement. θ_*0_ m*_ is the initial resting angle of MCP joint, θ_*i_ m*_ is the extended MCP joint angle upon wearing of the SECA in a unpressurized state, and *k*_*m*_ is the measured MCP joint stiffness. The hardware setup followed our previous work in Heung et al. (2020). The movement of SECA is controlled by input pneumatic pressure. A camera remains on top of the index finger for recording the passive MCP joint rotation.

To obtain the ground-truth value of MCP joint stiffness for verifying the accuracy of our method, we rebuilt the standard MCP joint stiffness measurement device. The device was demonstrated and confirmed accurate and effective previously by other research groups in Esteki and Mansour (1996) and Kuo and Deshpande (2012), as shown in Figure 2. The results from the measurement device is compared with the results obtained by our method for validation.

The device mainly consists of an arm rest, a finger splint, two load cells (Model 1021, Range 0-100 N, Arizon Inc., China), and a DC servomotor (RDS5160, Torque 60 kg.cm, DSservo Inc., China), as illustrated in Figure 2. The forearm of subjects is vertically clamped to the arm rest for maintaining the palm in vertical plane and the finger moving in horizontal plane. The arm rest can also prevent any lateral and rotational displacement of forearm during the test. The distal interphalangeal (DIP) and proximal interphalangeal (PIP) joints in the finger are clamped to the finger splint, such that the rotation of MCP joint can be coupled to the servomotor through the shaft. The sensor box that contains the two load cells is also placed on the shaft, allowing the tip of the finger splint to be embedded inside the box for measuring the force tangential to the circular path upon rotation of the finger.

#### MCP Stiffness Measurement

Prior to the measurement, the distance between the MCP joint and the tip of the finger splint is measured. Then, the MCP joint is passively moved by the servomotor at specific positions, as instructed by Esteki and Mansour (1996); Kamper and Rymer (2000), and Kuo and Deshpande (2012) and will be discussed in the following section. Upon each rotation, the passive tip force at the finger splint is measured and multiplied by the measured distance, resulting in the calculated passive MCP joint torque, as presented by

(5) $$\tau_{passive}=l_{fin}\times F_{passive}$$

which *l*_*fin*_ is the distance between the MCP joint and the tip of the finger splint (load cells), *F*_*passive*_ is the measured tangential force to the circular rotation of index finger, and τ_*passive*_ is the MCP joint torque. A classic double exponential function-based model developed for finger kinematics is further used to describe the relationship between the passive MCP joint moment and its corresponding joint angle (Esteki and Mansour, 1996; Silder et al., 2007; Deshpande et al., 2011), which is given by

(6) $$\tau_{passive}\left(\theta_{m}\right)=A\left(e^{-B\left(\theta_{m}-E\right)}-1\right)-C\left(e^{D\left(\theta_{m}-F\right)}-1\right)$$

that

$$\theta_{m}\in \left[\theta_{i\_m},\gamma \theta_{0\_m}\right],\gamma =0.7$$

which θ_*m*_ is the MCP joint angle (in degree), and *A* to *F* are the fitting parameters for the model. They are estimated using the Non-linear least squares (NLS) method in MATLAB based on the minimization of sum of squared differences (SSD) between the experimental values and the fitting model (Kuo and Deshpande, 2012). The MCP joint stiffness is obtained by taking derivative of equation (6) within the angle range of θ_*i_ m*_ and γθ_0_*m*_.

(7) $$k_{m}=\left|-CDe^{-D\left(\theta_{m}-F\right)}-ABe^{-B\left(\theta_{m}-E\right)}\right|$$

In equation (7), θ_*m*_ is substituted with the angle values located in the range between θ_*i_ m*_ and γθ_0_*m*_, such that stable stiffness values can be ensured and be not obscured by the torque increase upon closing to full flexion and extension of the MCP joint, which will be explained in the following section. Eventually, six joint angles are selected by portion, as suggested by Kamper and Rymer (2000), from the defined range and substituted into the stiffness equation (7). The average of all six calculated joint stiffness at different angle points is the final MCP joint stiffness measured by the device.

## Materials and Methods

### Subjects

Four subjects with chronic stroke who had demonstrated weak hand strength with moderate level of finger flexor spasticity was recruited. Four subjects with no neurological deficit from the laboratory also participated in the study to serve as the control. Both have given their informed consents. The clinical evaluation was registered to the Joint Chinese University of Hong Kong-New Territories East Cluster (CUHK-NTEC) Clinical Research Ethics Committee (Ref. ID: NCT03286309). Table 1 listed the demographic information of all the recruited subjects with stroke.

**TABLE 1:** Demographic information of the recruited subjects.

Subjects	Age	Gender	Stroke onset	Stroke type	Hemiplegic side	MAS	ARAT^3^	FMA-UE^4^
S1	65	Male	11 years	Hemorrhagic	Left	1+	25	27
S2	32	Male	48 months	Hemorrhagic	Left	2	3	16
S3	72	Female	62 months	Ischemic	Left	3	0	8
S4	59	Female	59 months	Ischemic	Left	1+	10	16
H1**^1^**	24	Male	–	–	– (Left)**^2^**	–	57	66
H2**^1^**	25	Male	–	–	– (Right)**^2^**	–	57	66
H3**^1^**	25	Male	–	–	– (Right)**^2^**	–	57	66
H4**^1^**	25	Male	–	–	– (Right)**^2^**	–	57	66

*^1^Subjects without neurological impairment as control group.*

*^2^MCP joints on the index fingers in the dominant hands were chosen for the assessment.*

*^3^ARAT (Action Research Arm Test) is a continuous measurement for the changes in upper limb function among hemiplegic subjects with no categorical cutoff scores. Full score is 57. Higher score indicates better upper limb performance.*

*^4^FMA-UE (Fugl-Meyer Assessment for Upper Extremity) is designed as performance-based impairment index to assess the motor function, balance, sensations, and joint functions in hemiplegic subjects with chronic stroke. In this work, FMA-UE refers only to the upper extremity motor function part with a total score of 66.*

### Experimental Configuration

The subjects are first examined with our stiffness evaluation method. Then, the subjects are further assessed with the replicated stiffness measurement device. Stiffness values obtained from two methods are compared with each other for validation.

#### SECA-Finger Modeling for Stiffness Evaluation

Index finger is selected as a stiffness indicator of the hand, similar to the approaches from different research groups (Kamper and Rymer, 2000; Heung et al., 2019, 2020). Examination of MCP joint stiffness in index finger is conducted from the pressure-angle relationship during the actuated flexion and extension of MCP joint with the SECA, as described in equation (1) – (4).

The SECA contains MCP segment for the actuation of MCP joint. It is attached to a hand base and secured to the index finger using Velcro Strap, as shown in Figure 2 already. To ensure result consistency, the posture of the upper limb needs to be defined during experiments, as already described in our previous work (Heung et al., 2020). The hand base of the soft robotic hand covers the wrist to maintain it in a neutral position (0° of flexion and extension) with respect to the forearm, while the forearm is also being held in its neutral position (0° of supination and pronation). The elbow is flexed 90° and supported on the desk. The subjects need to sit vertically to the table and relax the whole time during the experiment to ensure the collected MCP joint angle is solely due to the passive rotation driven by the SECA.

#### Ground-Truth Stiffness Evaluation With Standard Mechatronic Devices

**FIGURE 3:**
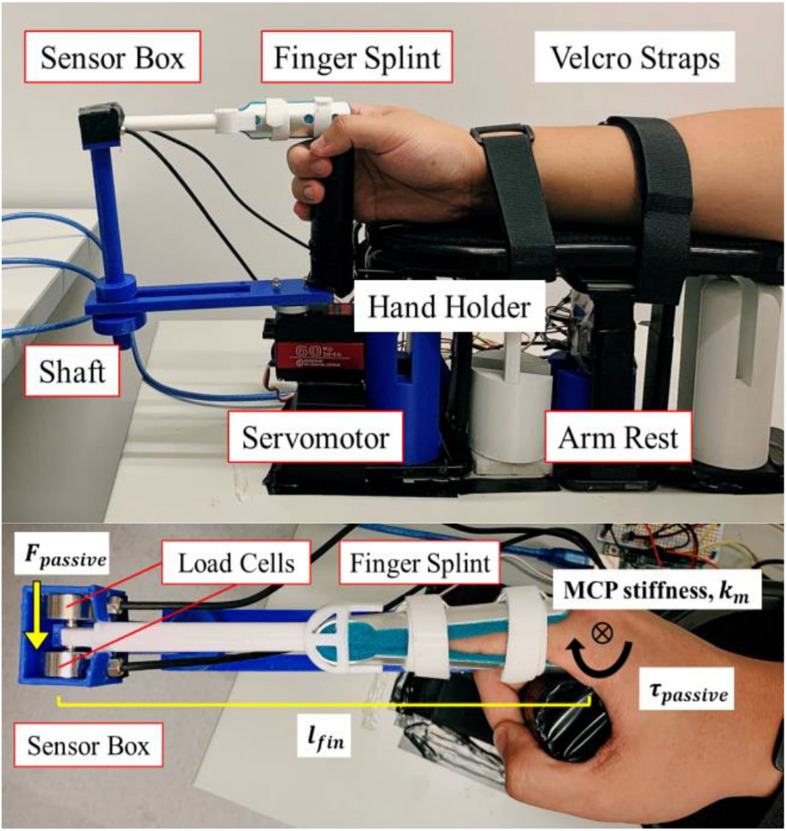
Stiffness measurement device replicated from Esteki and Mansour (1996) and Kuo and Deshpande (2012) for MCP joint stiffness testing in index finger. Two load cells are placed in the sensor box. The finger splint is embedded in the sensor box. The MCP joint is rotated by the DC servomotor. The forearm is secured on the arm rest, which includes Velcro straps and a hand holder.

Index finger is also selected as a stiffness indicator of the hand. The standard stiffness measurement device for measuring passive MCP joint angle and torque in index finger is replicated and used for obtaining the ground-truth stiffness values, as shown in Figure 3. Previous study already demonstrated the effectiveness of this device in quantifying the MCP joint stiffness in index finger (Esteki and Mansour, 1996) and (Kuo and Deshpande, 2012).

The DIP and PIP joints in the index finger are clamped by the finger splint, allowing solely the MCP joint to be rotated by the servomotor in horizontal plane. Passive extension force at the splint tip is measured by the load cells in the sensor box with the sampling rate of 80 Hz during passive rotation of MCP joint. The force is multiplied by the distance between the MCP joint and the splint tip, thereby obtaining the final values of passive MCP joint stiffness. The posture of upper limb follows the settings in the experiment using SECA, as elaborated previously. A hand holder is placed in the arm rest for holding the other four fingers in vertical plane. Velcro Straps are used to fix the forearm (0° of supination and pronation) and wrist (0° of flexion and extension) at the natural position, as illustrated in Figure 3. The subjects need to sit vertically with the elbow being flexed at 90° and supported on the desk. The subjects are required to stay relax the whole time as well to ensure the collected force is solely due to the passive extension driven by the servomotor.

### Protocol

A brief physical examination of MAS is first performed on assessing the flexor tone in the spastic finger. The degree of spasticity is assessed by a trained clinical assessor who is blind to the experimental condition. For the subjects with no neurological deficit, the examination of spasticity with MAS is not conducted, and the result can be classified as “no increase in flexor muscle tone” (MAS score = 0) (Kamper and Rymer, 2000). After confirming their MAS grades, the evaluation of MCP joint stiffness in the index fingers will be conducted.

Preparation procedures are required for the subjects prior to both experiments using our SECA and the standard measurement device. To maintain the assessed muscle tone being consistent and stably presented on subjects in two tests, they are instructed to sit vertically for 30 min with the elbow at 90° and supported on the desk to relax the forearm muscles prior to each test. Any passive mobilization to extend the fingers, or any generated movement (either passive or active) on the fingers that will greatly alter the muscle tone is not allowed during the relaxation time. The 30 min resting time before the spasticity measurement was suggested by Kakebeeke et al. (2005) to minimize the effect of any movement or mobilization taken before the test.

In the first stiffness test using the SECA, unreliable MCP stiffness results due to model singularity (i.e., small difference between the resting angle of MCP joint and the current bending angle of MCP joint) and the extra passive MCP joint resistance provided by the joint CLC upon closing to full flexion or extension can be excluded by setting the angle range between θ_*i_ m*_ and γθ_0_*m*_(γ = 0.7). Stable stiffness results can be ensured within the defined angle range. Also, only three repetitions are performed to measure the passive MCP joint angle, as it has been suggested that short-term effect of any stretch, e.g., loosening the joints and reducing the muscle tone, may occur if more than three times (Charalambous, 2014; Levine, 2018).

During the measurement, the SECA is placed on the index finger in an unpressurized state. Pressure is then increased step by step from zero by 20 kPa one at a time to move the MCP joint to different angles, which the measured angles and the corresponding pressure values are recorded. The SECA holds the MCP joint at each position for 30 s to ensure stable angle values can be collected (Esteki and Mansour, 1996). As soon as the MCP angle reaches its upper limit γθ_0_*m*_, the MCP joint will slowly (2° per second) return to the extended position θ_*i_ m*_ with the SECA, such that the dynamics of SECA-Finger model can be ignored. This whole process is defined as one complete repetition. Upon completion of all three repetitions, three MCP joint angle values would have been recorded at each specific pressure input, e.g., at 0, 20 kPa, etc., respectively. Average MCP joint angle at each pressure input is taken from the three measured angle values and substituted into the static model in equation (1) – (4) to obtain the measured stiffness value at each specific pressure input. Eventually, the final MCP joint stiffness is determined from the average of all measured stiffness values.

On the other hand, the standard stiffness measurement device is used in the second stiffness test. Previous study used the measurement device to rotate the MCP joint from the flexed position (50°) to the fully extended position (−40°), i.e., ROM of 90°, and obtain the passive MCP stiffness values for this overall range (Kamper et al., 2006). With sufficient data collected from this range, a linear pattern of stiffness values could be observed in the range from 0° to 50°. To reduce any potential discomfort due to full extension of the joint (−40° to 0°), we adjusted the testing ROM from the range of −40° to 50° to the range of 0° to 90°. The device therefore rotates the MCP joint step by step from 90° (flexion) to 0° (extension) by 10° one at a time. The speed during each rotation is 2° per second, such that the dynamics of fingers can be ignored. The servomotor holds the MCP joint at each position for 30 s to ensure stable tip force values can be collected (Esteki and Mansour, 1996). As soon as the MCP joint angle reaches extension (0°), the MCP joint will slowly (2° per second) return to the flexed position (90°) with the servomotor. This whole process is defined as one complete repetition. Another reason of rotating the joint with the device in one repetition is that we want to further demonstrate the existence of extra passive MCP joint resistance due to the joint CLC in full flexion or extension (Kuo and Deshpande, 2012), which further supported to define the angle range. Besides, during the fitting of the double exponential function-based model for describing finger kinematics, it is suggested that torque values measured from full ROM are required and necessary, as mentioned previously (Kuo and Deshpande, 2012).

Similar to the stiffness test using SECA, upon completion of all three repetitions, three measured MCP joint torque values would have been recorded at each specific joint position, e.g., at 90°, 80°, etc., respectively. Average MCP joint torque at each joint position is taken from the three measured torque values and fitted into the double exponential function-based model in equation (6) for getting the parameters. The parameters are then substituted into equation (7) for the stiffness equation. Six joint angles are selected by portion, as suggested by Esteki and Mansour (1996); Kamper and Rymer (2000), and (Kuo and Deshpande, 2012), from the defined angle range between θ_*i_ m*_ and γθ_0_*m*_(γ = 0.7) and substituted into the stiffness equation (7). Eventually, the average of all six calculated joint stiffness is the final MCP joint stiffness measured by the stiffness measurement device. Throughout all the tests, subjects did not indicate any discomfort and pain upon the stretch of their MCP joints.

## Experimental Results

### Stiffness From the SECA

**FIGURE 4:**
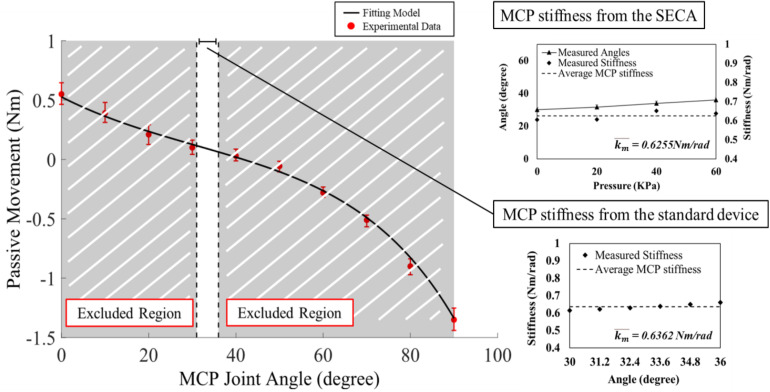
Examples of the experimental data and model fitting results for subject S3 with chronic stroke with MAS = 3. The torque and angle of the MCP joint in S3 is collected from the ROM of 0° to 90° for model fitting. Results of MCP joint stiffness in S3 are presented by two different estimation methods. Using the SECA, pressure is increased by 20 kPa until the angle reaches the upper limit. Using the standard device, six joint angles are selected by portion in the angle range for estimation of MCP joint stiffness. Error bar presents the different between the maximum and minimum measured stiffness values. Final joint stiffness is taken by the average of all measured values.

Quantification of MCP joint stiffness is conducted. Pressures of 0, 20, 40, and 60 kPa are applied to the SECA. Bending angles of MCP segment, and the corresponding actuation pressures, are recorded for the calculation of stiffness values using equation (2). To avoid singularity, it should be noted again that the maximum MCP joint angle cannot exceed their pre-defined upper limits. Table 2 shows the MCP joint stiffness of all eight subjects measured by the SECA. The data from subject S1 with chronic stroke and subject H1 with no neurological deficit is also plotted in Figure 4 as well as an example to demonstrate the stiffness evaluation for S1 and H1, which the stiffness of 0.0809 Nm/rad and 0.0316 Nm/rad is obtained respectively from the average of different measured stiffness values at different pressure values.

**TABLE 2:** Summarization of MCP joint stiffness characteristic with different measurement approaches.

	MAS	^θ_0_*m*_^ ^1^	[^θ_*i*_*m*_,γθ_0_*m*_^]^2^	Ground-truth ^*k_m (Nm/rad)*3^	*k*_*m*_ from SECA ^*(Nm/rad)4*^	Difference
S1	1+	54°	[18°, 38°]	0.0849 (±0.0158)	0.0809 (±0.0025)	0.004 (±0.0066)
S2	2	64°	[34°, 45°]	0.5217 (±0.0436)	0.5011 (±0.0408)	0.021 (±0.0271)
S3	3	51°	[30°, 36°]	0.6362 (±0.0173)	0.6255 (±0.0231)	0.011 (±0.0135)
S4	1+	35°	[13°, 25°]	0.0930 (±0.0099)	0.0857 (±0.0106)	0.007 (±0.0067)
H1	–	45°	[10°, 32°]	0.0377 (±0.0032)	0.0316 (±0.0003)	0.006 (±0.0013)
H2	–	46°	[9°, 32°]	0.0328 (±0.0183)	0.0277 (±0.0004)	0.005 (±0.0077)
H3	–	40°	[10°, 28°]	0.0466 (±0.0057)	0.0408 (±0.0006)	0.006 (±0.0037)
H4	–	58°	[11°, 41°]	0.0393 (±0.0059)	0.0295 (±0.0026)	0.010 (±0.0027)

*^1^The resting MCP joint angle upon wearing of the SECA onto the index finger at zero pressure.*

*^2^The angle range for the calculation of MCP joint stiffness with the standard stiffness measurement device and our SECA.*

*^3^MCP joint stiffness measured by the standard stiffness measurement device.*

*^4^MCP joint stiffness measured by our SECA.*

### Stiffness From the Standard Stiffness Measurement Device

Table 2 presents the MCP joint stiffness of all eight subjects measured by the standard stiffness measurement device. The data from subject S1 and with chronic stroke and subject H1 with no neurological deficit is also plotted in Figure 4 as an example to demonstrate the stiffness evaluation for S1 and H1, which the stiffness of 0.0849 Nm/rad and 0.0377 Nm/rad is obtained respectively from the average of different measured stiffness values at different joint angles. In Figure 4, it is worth to note that the MCP joint is stretched in full range to obtain complete set of torque values with respect to the joint positions for the fitting of the double exponential model. The calibrated model parameters are then substituted into equation (7) for the measured stiffness values at different joint angles. However, to minimize the effect of the CLC to the MCP joint stiffness, only the joint angles within the range listed in Table 2 are adopted to stiffness evaluation. Table 3 also shows the fitting parameters for the double exponential model and the MCP joint stiffness equation.

**TABLE 3:** Fitting model parameters.

	*A*	*B*	*C*	*D*	*E*	*F*	*R*^2^
S1	0.005	0.073	0.130	0.045	21.88	63.58	0.88
S2	0.006	0.050	0.065	0.041	30.75	10.64	0.91
S3	0.015	0.028	0.230	0.038	21.79	68.11	0.88
S4	0.030	0.035	0.082	0.044	80.46	25.33	0.89
H1	0.001	0.097	0.100	0.032	19.23	75.34	0.91
H2	0.006	0.077	0.016	0.081	17.33	60.25	0.90
H3	0.005	0.080	0.038	0.046	17.57	55.16	0.89
H4	0.003	0.081	0.055	0.042	20.81	67.87	0.93

### Comparison Between Two Approaches

The results from two different approaches are similar with each other. Upon the increase of muscle tone (spasticity), the MCP joint stiffness further increases. Only a maximum stiffness difference of 0.021 Nm/rad is observed in subject S2 with chronic stroke, and minimum difference of 0.004 Nm/rad in subject S4 with chronic stroke. From our data, it is also observed that the stiffness from our SECA would be slightly smaller than the actual joint stiffness. Here, we believe that the stiffness evaluation method using our SECA is feasible for finger joint stiffness quantification during any rehabilitation training. It would be possible to extend the quantification to the proximal interphalangeal (PIP) or distal interphalangeal joints (DIP) joints as well using our SECA, which has been hard to be accomplished by the standard stiffness measurement device.

## Discussion

Spasticity is a well-known symptom seen in subjects after suffering a stroke. Hand function is severely affected due to the excessive flexor tone in muscles. The purpose of this study was to validate the compatibility of our stiffness quantification method in clinical practices. We quantified the passive MCP joint stiffness in subjects with chronic stroke and no neurological deficit. We also investigated the difference of stiffness results obtained from our SECA and the standard stiffness measurement device. The results between using our approach with the SECA and the standard device were consistent, which only a difference of 0.0044 Nm/rad was observed in the MCP stiffness. The results were also supportive when comparing with MAS.

The passive MCP stiffness evaluation was conducted with the replicated standard device to collect the passive joint torque and ROM in each subject. The double exponential function-based model fitted well with the collected finger kinematic data, which the performance was similar to previous research (Knutson et al., 2000; Kuo and Deshpande, 2012). From the results obtained from existing research and using the standard stiffness measurement device, it was disclosed that the measured passive MCP joint extension torque significantly increased as soon as the joint angle was approaching to its limit of rotation. All the subjects exhibited larger joint stiffness upon closing to full flexion or extension of MCP joints, which is suggested due to the extra resistance provided by the joint CLC apart from the finger flexors for maintaining finger joint stability (Kuo and Deshpande, 2012). In spasticity assessments, the assessors would also leave the fingers some space without fully extending them because of safety concerns and not creating discomfort. Passive resistance due to CLC would not be assessed and considered as part of the muscle tone at all. Therefore, ROM of MCP joints during stiffness evaluation would be restricted around the resting position based on the range defined in equation (3) and (4). This would mainly assess the MCP stiffness due to finger flexor tone and the values would tend to be stable within the defined range around the resting position.

Common clinical examinations of spasticity include the Ashworth Scale (AS) and Tardieu Scale (TS). To further quantify the MCP stiffness after stroke, there is not a lot of studies conducted the quantify the stiffness of spastic finger joint to the best of our knowledge. We have concluded the results from some existing conclusive studies and presented in Table 4. Results of MCP stiffness values from our and existing research reflected the possible similar joint properties between subjects having MAS of 0, 1, and 1+ and subjects with no neurological deficit. For subjects with no neurological deficit, depending on their body characteristics, e.g., age, weight, gender, muscle strength, etc. (Dionysian et al., 2005), their MCP joint stiffness could vary greatly, e.g., from around 0.02 Nm/rad to 0.2 Nm/rad, as reviewed in Table 4, among each individual. In our four subjects with no neurological deficit, their MCP stiffness ranged from 0.03 Nm/rad to 0.05 Nm/rad, which was also located in the range in Table 4. For subjects with chronic stroke having MAS of 0, 1, and 1+, their MCP joint stiffness was observed to be closed to some subjects with no neurological deficit, as comparing the 1+ grade and the 1–3 grade range with subjects with no neurological deficit in Table 4. In our subjects with chronic stroke with MAS of 1+ grade, the MCP stiffness was around 0.09 Nm/rad, which was also located in the range of MAS between 1 and 3, e.g., from around 0.09 Nm/rad to 1.13 Nm/rad, and closed to the value of 1+ grade in Table 4. The stiffness value was also located in the range of subjects with no neurological deficit, e.g., from around 0.02 Nm/rad to 0.2 Nm/rad. Indeed, more cautions should be taken to assess subjects with mild spasticity as their finger characteristics in terms of post-stroke stiffness would be similar. One possible way to optimize the assessment in an objective manner would be to classify the joint stiffness range for the mapping of each MAS grade with more test subjects with chronic stroke in the future (Pandyan et al., 1999; Towles et al., 2010; Brokaw et al., 2011; Zakaria et al., 2015).

**TABLE 4:** Existing studies about the quantification of MCP joint stiffness on both subjects with chronic stroke and no neurological deficit.

Test subjects	Studies	MAS	Estimated MCP stiffness *k*_*m*_ (*Nm/rad*)
Subjects with chronic stroke	Towles et al., 2010	1 – 3^1^	∼ 0.09337 to ∼1.12894 (*n* = 12)
	Kamper and Rymer, 2000	3	∼0.60733 to ∼ 0.88236 (*n* = 3)
		2	∼0.22345 to ∼ 0.55004 (*n* = 6)
	Brokaw et al., 2011	2	∼0.53476 (*n* = 1)
		1+	∼0.14323 (*n* = 1)
Subjects with no neurological deficit	Kuo and Deshpande, 2012	–	∼0.01578 to ∼ 0.21046 (*n* = 10)^2^
	Esteki and Mansour, 1996	–	∼0.02535 to ∼0.05792 (*n* = 3)^2^

*^1^Joint stiffness values were organized as a whole group without classification with respect to different MAS.*

*^2^Values were estimated in the angle region between 0° and resting angle θ_*0_ m*_. Stiffness increase when approaching full MCP joint flexion and extension was not considered.*

As soon as excessive muscle tone further increases in finger flexors, larger MCP stiffness values could eventually be observed. For subjects with chronic stroke having MAS of 2 and 3, their MCP joint stiffness was observed to be much larger than that of subjects with no neurological deficit, e.g., from around 0.22 Nm/rad to 1.12 Nm/rad in Table 4. In our subject with chronic stroke having the MAS of 2 grade, the MCP stiffness was around 0.5 Nm/rad, which was higher than that of subjects having lower MAS grade. Stiffness values for subjects having MAS higher than 2 would be higher than subjects having lower MAS and subjects with no neurological deficit. For subjects having MAS of the 3 grade, stiffness values could be up to 1 Nm/rad and obviously higher than that of other subjects with less spasticity. From the MCP stiffness results, we might also know that assessors would be easier to distinguish the condition of subjects having stronger spasticity (MAS ≥ 2) comparing with subjects having less spasticity (MAS < 2), as more marked increase of resistance against passive joint movement would be more easier for assessors to recognize during assessment. Overall, larger stiffness results would be observed in subjects with higher MAS grade. Values of their MCP joint stiffness in both subjects with chronic stroke and no neurological deficit were also found to be near the stiffness ranges presented by previous studies, as summarized in Tables 3, 4 already. This shows the potentials of our quantification method based on the SECA-Finger model and integrating it into hand rehabilitation training in future studies for observing the possible recovery process of finger stiffness.

This work explored the difference of passive MCP joint stiffness among subjects who experienced a stroke or remain neurologically intact. Experimental results provided some evidence of quantifying the MCP stiffness using our SECA. In the future, our proposed methods could be applied to our rehabilitation robotic hand that reflects the stiffness for individual finger. With the stiffness information, optimal training tasks might be planned for each individual with stroke depending on the finger stiffness condition. Therapeutic progress might also be indicated in detail to motivate patients for achieving better improvement during rehabilitation training.

## Data Availability Statement

The raw data supporting the conclusions of this article will be made available by the authors, without undue reservation.

## Ethics Statement

The studies involving human participants were reviewed and approved by Joint Chinese University of Hong Kong-New Territories East Cluster (CUHK-NTEC) Clinical Research Ethics Committee (Ref. ID: NCT03286309). The patients/participants provided their written informed consent to participate in this study. Written informed consent was obtained from the individual(s) for the publication of any potentially identifiable images or data included in this article.

## Author Contributions

XS and HH developed the mathematical theories, designed the experimental devices and experimental protocols, implemented the data collection software, performed the experiments, analyzed the experimental results, and participated in manuscript preparation. HH, XS, and ZT performed the experiments, contributed to discussions, analysis, and participated in manuscript revisions. KT and ZL supervised the project, contributed to discussions, analysis, and participated in manuscript revisions. All the authors have approved the submitted manuscript.

## Conflict of Interest

The authors declare that the research was conducted in the absence of any commercial or financial relationships that could be construed as a potential conflict of interest.
